# A Systematic Review of the Impact of Electromagnetic Waves on Living Beings

**DOI:** 10.7759/cureus.90355

**Published:** 2025-08-17

**Authors:** Saliba Danho, Juan Felipe Escobar Huertas, Wolfgang I Schoellhorn

**Affiliations:** 1 Department of Movement Science and Training/Biomedical Engineering, Johannes Gutenberg University Mainz, Mainz, DEU; 2 Department of Biomedical Engineering, Universidad Nacional de Colombia, Bogota, COL; 3 Department of Movement Science and Training, Johannes Gutenberg University Mainz, Mainz, DEU

**Keywords:** biological systems, electromagnetic fields, emf exposure, environmental exposure, frequency-dependent effects, genotoxicity, health effects, oxidative stress, physiological responses, systematic review

## Abstract

The effects of electromagnetic fields (EMFs) have been extensively debated among researchers and the public, with their critical consequences often dismissed or deemed unscientific. In light of this, we conducted this systematic review that extensively focuses on the detrimental effects of EMFs on living organisms.

A comprehensive and systematic literature search was performed on various electronic databases, including PubMed, Scopus, and the Cochrane Library, using Preferred Reporting Items for Systematic Reviews and Meta-analyses (PRISMA) guidelines. This review concentrates on experimental studies published between 2017 and 2024 that investigated physiological or behavioral responses to EMF exposure, with particular attention given to those reporting harmful or concerning effects. Documented impacts include effects on humans, animals, and plants, targeting various cell types (e.g., blood, cancer, thyroid, cochlea), genotoxicity, cardiovascular parameters (e.g., heart rate, blood pressure), male fertility (e.g., testes, sperm), neuronal brain activity, and photosynthesis in plants. Methodological quality was assessed using established bias assessment tools, and certainty of evidence was evaluated according to the GRADE framework.

After screening, 24 studies were included in the present review; five studies were non-randomized and involved humans, seven studies were in vitro, and 12 studies were conducted on animals. The findings demonstrated that EMFs negatively affect a wide array of biological systems of living organisms, including mechanisms of oxidative stress, inflammatory responses, and disruptions in cellular, physiological, and ecological processes. Most of the included studies showed a moderate to high risk of bias, which contributed to a lower overall certainty of the evidence.

These findings underscore the significant health and environmental risks associated with rising exposure levels of EMF, highlighting the urgent need for strategies to mitigate the risks. Despite these valuable insights, significant research gaps persist because the long-term effects of EMF exposure, especially on human populations, remain poorly understood and warrant further investigation and targeted mitigation strategies.

## Introduction and background

Background

The health and environmental impact of electromagnetic fields (EMFs) has long been scientifically contested. EMFs can be broadly divided into ionizing and non-ionizing types, and this review focuses on non-ionizing EMFs. Unlike ionizing radiation (e.g., X-rays, gamma rays), non-ionizing EMFs lack sufficient photon energy to break molecular bonds or directly damage DNA, and their primary interactions with biological systems occur through mechanisms such as induced currents or tissue heating. These can produce both thermal effects, related to tissue heating, and non-thermal effects, which occur without a measurable temperature increase. The biological impact often depends on the frequency, making it a key factor in exposure assessment. Since the mid-20th century, the rapid expansion of wireless technologies, such as Wi-Fi (2.4-5 GHz) and mobile networks (700 MHz to 2.6 GHz), has substantially increased public exposure to EMFs [1-3].

While some studies report neutral or even therapeutic effects of these fields, an increasing number of studies highlight adverse outcomes such as oxidative stress, genotoxicity, endocrine disruption, and reproductive impairments [4-6]. It is important to note that such classifications, whether the effects are “adverse” or “beneficial,” are often context-dependent. EMF effects may differ depending on exposure duration, frequency, and the affected biological system. However, heterogeneous methodologies, inconsistent exposure settings, and non-standardized endpoints complicate interpretation and comparison [7,8]. Consequently, the clinical significance and reproducibility of reported biological effects, particularly for low-frequency EMFs, remain uncertain, with many findings still debated in terms of their mechanistic plausibility and consistency across studies.

Problem statement

Such controversy is not new. Historically, many EMF-related technologies have been met with skepticism. During the ‘War of the Currents’, in the late 19th century, Thomas Edison publicly warned of the dangers of alternating current (AC) and staged dramatic demonstrations to influence public perception [9,10]. Similar fears surrounded the introduction of microwave ovens [11]. Though sometimes exaggerated, such skepticism often triggered important safety research, standard-setting, and regulation. In EMF research, critical scrutiny continues to play a constructive role by promoting evidence-based guidelines and public health protection [12]. Importantly, skepticism should not be dismissed as obstructive - it fosters scientific progress by encouraging methodological rigor, balanced risk assessment, and caution in interpretation, particularly in complex interdisciplinary fields like EMF research [13,14].

Given this context, systematic reviews are essential, as they help make sense of scattered findings. They also help bring structure and order to a complex area, along with helping minimize bias, evaluate study quality, and identify research gaps [15,16]. This review evaluates experimental studies published between 2017 and 2024 that report detrimental effects of artificial non-ionizing EMFs on humans, animals, and plants. By synthesizing recent findings, it aims to guide risk assessment and inform future research. Yet interpretation remains limited by study design and context. Overgeneralization can lead to misleading conclusions, a core concern in the reproducibility crisis and scientific generalization debates [17,18]. Science is context-dependent and not universally transferable. Systematic reviews do not yield definitive claims but help detect patterns and knowledge gaps. As highlighted by the philosophy of science, generalizations are only valid if underlying mechanisms remain stable across conditions [19,20].

Objectives

EMFs are typically classified as low-frequency (0-300 Hz, e.g., power lines, appliances) or high-frequency (10 MHz-300 GHz, e.g., mobile phones, microwave ovens) [21]. Low-frequency fields may induce weak currents in tissues, affecting neural and muscular systems [22,23], while high-frequency fields are linked to both thermal and non-thermal effects, including oxidative stress, calcium overload, and cellular dysfunction [5]. Fifth-generation (5G) EMFs penetrate tissue to varying depths depending on frequency and exposure parameters [24,25]. Natural EMFs, such as Schumann resonances and subtle geomagnetic shifts, have long been linked to biological timing systems. When these patterns are disturbed - say, during heightened solar activity - it may lead to measurable shifts in circadian stability [26,27].

Despite the technological benefits, public concern persists. Advances in dosimetry have improved exposure quantification [28], yet challenges remain due to overlapping frequencies, modulation types, and regional grid differences (e.g., 50 Hz in Europe vs. 60 Hz in the USA) [7,8]. Accurate exposure assessment requires precise quantification of field strength, magnetic flux density, and power density [29]. Scientific opinions remain divided. Some findings suggest beneficial or neutral effects, while others report harm; these differences may stem from study design, aims, or funding bias [30]. For example, during 1980-2002, over 200 studies examined the health impacts of power line EMFs, with 60% reporting no harm and 40% indicating negative effects [31-33]. EMFs may affect reproductive health, hormonal balance, and embryonic development, depending on intensity, frequency, waveform, and biological system involved [6,34,35]. Some recent studies point to possible cumulative effects from long-term exposure to high-frequency EMFs, leaving open questions that have yet to be fully explored.

Major health organizations, including the World Health Organization (WHO) and the International Commission on Non-Ionizing Radiation Protection (ICNIRP), generally state that there is no conclusive evidence of adverse health effects below established exposure limits, while acknowledging that certain experimental results warrant further investigation. This review addresses the following question: What are the potential effects of EMF on living organisms, based on current evidence? The focus includes both physiological and psychological endpoints across diverse exposure types - from environmental radiation and power infrastructure to wireless communication systems - offering a comprehensive overview. To contextualize the discussion, Table 1 summarizes the main EMF types, including frequency, source, and associated biological systems.

**Table 1:** Overview of EMF types ^*^According to electromagnetic theory, static electric and magnetic fields are considered distinct phenomena. EMFs in the strict sense occur only when charges or fields vary with time (alternating current) EMF: electromagnetic field

Field type	Frequency range	Typical intensity	Examples/sources	Biological systems affected
Static magnetic field^*^	0 Hz	~50 µT (Earth), up to several Tesla in MRI	Earth’s geomagnetic field, MRI magnets, and permanent magnets	Brain orientation, plant growth direction, and the circadian system
Static electric field^*^	0 Hz	Up to several kV/m	Electrostatic charges, high-voltage DC lines, and synthetic materials	Skin potential, electrostatic perception, surface charge accumulation
Low-frequency EMF	0–300 Hz	0.1–10 µT; 1–10 V/m	Power lines, household devices	Nervous, muscular, and cardiovascular systems
Intermediate-frequency EMF	300 Hz–10 MHz	1–100 V/m (varies), µT-range magnetically	Inductive cooking, video displays, and RFID	Cellular stress, organ-level functions
High-frequency EMF	10 MHz–300 GHz	0.1–10 V/m (indoor); >100 V/m (near-field); µT/mT possible	Microwave oven (~2.45 GHz), FM radio (~100 MHz), TV (~500 MHz), Wi-Fi, radar	Endocrine, reproductive, oxidative systems
Natural dynamic EMFs	~7.8 Hz (Schumann), 14.3 Hz, etc.	<1 µT; electric component usually negligible	Schumann resonances, solar magnetic fluctuations, and atmospheric electricity	Circadian regulation, neuronal oscillation

## Review

Methods

Study Design

This systematic review was performed based on the 27-item Preferred Reporting Items for Systematic Reviews and Meta-analyses (PRISMA) guidelines [15]. This review was registered in PROSPERO with registration number CRD420251067528.

Selection Criteria

Standardized selection criteria were developed using the PICOS (Population, Intervention, Comparison, Outcome, Study Type/Setting) framework in this review. A well-known method for organizing literature reviews to successfully answer research questions is the PICOS framework [16].

Population: living organisms; Intervention: exposure to artificial electromagnetic waves in the frequency range 0 to 300 GHz, including low and high frequencies, with varying intensities (typically 0.1-10 V/m or higher); Comparison: none; Outcome: negative effects and influences; Study type: empirical studies, including randomized control trials (RCTs) and non-RCTs.

In addition, only those studies published between 2017 and 2024 were considered, which is particularly important in the context of current mobile network expansions that have decreased coverage’s “white gaps.” Furthermore, only studies published in English or the German language were included.

Similarly, exclusion criteria were also applied, and studies performed on non-living organisms, and studies on mobile phone frequencies associated with 5G were excluded since 5G research often involves unique frequency ranges, typically in the mm wave spectrum, modulation characteristics, and exposure scenarios that differ significantly from the broader range of EMW sources. Studies that reported a positive impact of EMWs, editorials, abstracts, proceeding papers, presentations, protocols, and reviews published in non-English or German languages were excluded.

Search Strategy

The literature search was conducted using MEDLINE, Scopus, and the Cochrane Library using keywords like electromagnetic waves, electromagnetic field, EMF, electromagnetic radiations, Schumann frequencies, Schumann resonance, living beings, living organisms, humans, animals, cells, biological systems, negative effects, negative outcome, detrimental impact, and adverse effects. These search terms were combined using Boolean operators (OR, AND); the detailed search strategy is presented in the Appendices.

Screening Process for Study Selection

**Figure 1:**
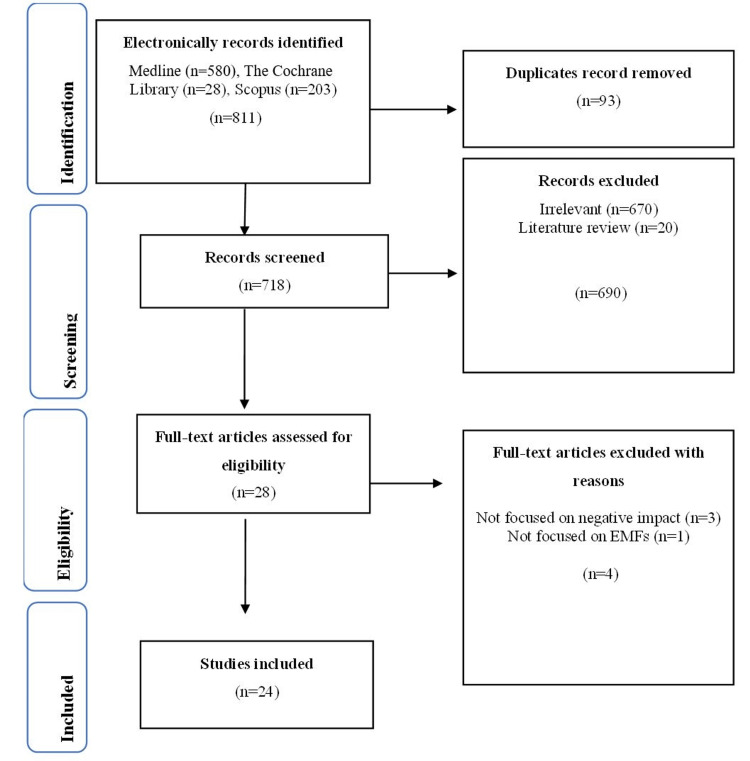
PRISMA flow chart depicting the selection of studies PRISMA: Preferred Reporting Items for Systematic Reviews and Meta-analyses

A PRISMA flowchart based on four phases was used for the selection of studies by two independent reviewers (Figure 1). Disagreements at any screening stage were resolved by discussion and consensus between the two reviewers; no third adjudicator was required. In the first phase, 811 studies were identified from electronic databases and transferred to EndNote X9 referencing software to exclude 93 duplicate studies. In the second phase, 718 studies were reviewed and evaluated to determine whether they adhered to the aim of the study. Twenty-eight studies relevant to our review moved to the third phase, while the remaining 690 studies were excluded due to irrelevance to the research question, incomplete data, or lacking methodological quality. In the third phase, a full-text assessment of 28 studies was performed, and selection criteria were strictly followed for the inclusion of the studies in the present review. Twenty-four studies that fulfilled our selection criteria were moved to the last phase, while the remaining four studies were excluded due to inconsistent outcome reporting, insufficient exposure details, or use of animal models not aligned with the inclusion criteria. In the inclusion phase, the 24 studies were further analyzed.

Data Extraction

A predefined data collection sheet was used by two independent reviewers (the authors Saliba Danho and Juan Escobar) for the extraction of the data. The extracted parameters included study ID, classification, study design, sample size, frequency, field strength, magnetic flux density, exposure conditions, duration, statistical analysis, outcome measures, effect size, key findings, relevance to humans, strengths and limitations, conclusion, evidence grade, and study funding source.

Methodological Quality - Assessment Approach

To ensure the inclusion of high-quality studies, all included studies were assessed using standardized quality evaluation tools corresponding to their study design. Risk of Bias in Non-Randomized Studies of Interventions (ROBINS-I) was used for non-RCTs; Robvis, a web-based application, was used to highlight the outcomes of non-RCTs [36, while QUIN was employed for in vitro studies [37,38]. This assessment tool has 12 items, and each study was evaluated according to these items and rated as yes (allocating 1-2 points), no with 0 points, or not applicable [39]. Later, each study was rated according to the point response. Study scores <50% were considered to have a high risk of bias (RoB), those with 50-70% to have medium RoB, and those with scores >70% to have low RoB [39]. Meanwhile, animal studies were evaluated according to the SYRCLE guidelines introduced by the Systematic Review Center for Laboratory Animals and Experimentation. This assessment tool is based on six domains: selection, performance, detection, attrition, reporting, and others [40]. These tools were selected for their robustness in identifying potential sources of bias and their alignment with the methodological rigor required for this review. The quality assessment process ensured that studies with "Low Risk" or "High Quality" ratings contributed more significantly to the synthesis.

Data Analysis

A narrative synthesis was performed to systematically summarize the findings. Data were extracted from each study and organized into structured tables highlighting key characteristics of the studies. The synthesis focused on the identification of consistent patterns and differences across studies. In addition, the strengths, limitations, and quality of evidence of each study were also documented. All reported exposure metrics (e.g., electric field strength, magnetic flux density, specific absorption rate (SAR)) were expressed in SI units (V/m, µT, W/kg) whenever conversion was possible. Where original studies reported non-SI units (e.g., mW/cm²), these values were retained to preserve data integrity, with units stated explicitly. 

To contextualize exposure magnitude, reported SAR or field values were qualitatively compared with relevant ICNIRP guideline reference levels. We did not impute missing SAR from other metrics and performed no cross-study normalization beyond unit conversions. No meta-analysis or meta-regression was conducted due to the considerable heterogeneity of study designs, exposure parameters, and reported outcomes. Instead, a qualitative (narrative) synthesis was applied to systematically summarize and compare the findings across studies.

Certainty of Evidence

The Grading, Reporting, Assessment, Development, and Evaluation (GRADE) framework was used to analyze the certainty of evidence; it is a structured and reproducible framework based on certain domains, like risk of bias, inconsistency, indirectness, and publication bias. Each outcome was evaluated according to these domains and rated as low, moderate, or high certainty of evidence [41].

Methodological Quality Assessment

Non-randomized human studies: Five studies followed observational study designs, including cross-sectional, cohort, and prospective approaches. Three of them - Al-Bayyari [42], Boileau et al. [43], and Kösek et al. [44] - were rated as having a low overall risk of bias. These studies applied well-defined exposure conditions, outcome measures, and participant selection strategies, reducing the likelihood of systematic error. Two studies - Szemerszky et al. [45] and Yahya et al. [46] - showed a higher risk of bias in several domains, such as confounding, participant selection, and outcome assessment. For instance, Yahya et al. [46] relied on short-term ECG recordings without controlling for external factors (e.g., caffeine intake, circadian rhythms), potentially inflating the EMF effect on heart rate variability. Szemerszky [45] used self-reported questionnaires without objective exposure validation, increasing reporting and selection bias.

**Figure 2:**
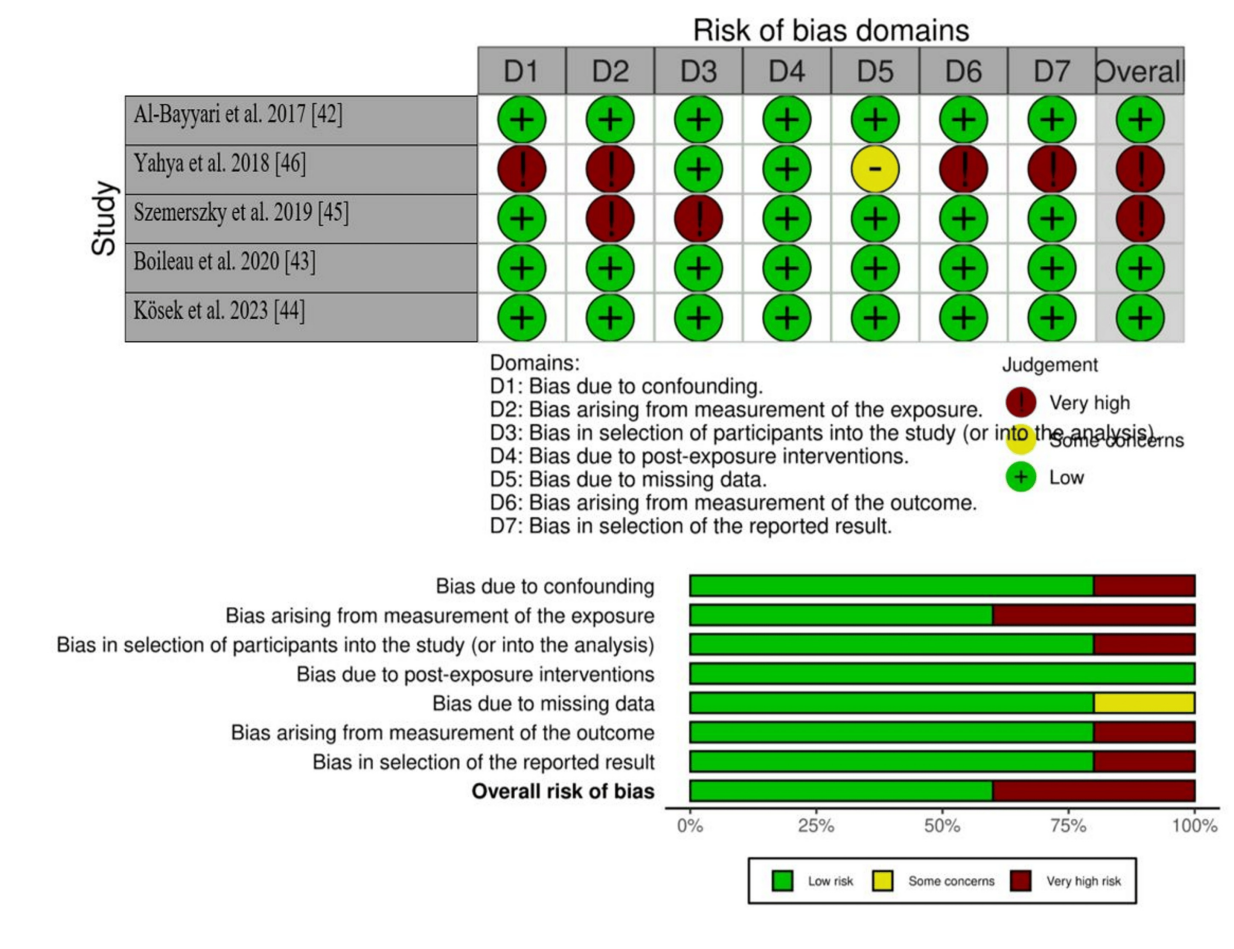
Risk of bias in non-randomized human studies assessed using the ROBINS-I tool

The assessed risk-of-bias domains included confounding, exposure measurement, participant selection, post-exposure interventions, missing data, outcome measurement, and selective reporting. These criteria and judgments are visualized in Figure 2 and should be considered when interpreting findings from non-randomized human studies.

In vitro studies: Most of the studies achieved scores <70% with a moderate risk of bias in the domains of sample size calculation, randomization, and blinding [47-52]. One study had a low risk of bias as it had a score of>70% [53], as summarized in Table 2.

**Table 2:** Methodological quality assessment of in vitro studies using QUIN assessment tool

Study	Aims/objectives	Sample size calculation	Comparison group	Methodology explanation	Operator details	Randomization	Method of measurement of outcome	Outcome assessor details	Blinding	Statistical analysis	Presentation of results	Total points	% age	Status
Chu et al. [47]	2	0	2	2	NA	0	2	NA	0	2	2	12	66.66	Moderate
Górski et al. [48]	2	0	2	2	NA	0	2	NA	0	2	2	12	66.66	Moderate
Górski et al. [49]	2	0	2	2	NA	0	2	NA	0	2	2	12	66.66	Moderate
Lefebvre et al. [50]	2	0	2	2	NA	0	2	NA	0	2	2	12	66.66	Moderate
López-Martín et al. [51]	2	0	2	2	NA	0	2	NA	0	2	2	12	66.66	Moderate
Sukhov et al. [52]	2	0	2	2	NA	0	2	NA	0	2	2	12	66.66	Moderate
Echchgadda et al. [53]	2	0	2	2	NA	1	2	NA	0	2	2	13	72.22	Low

Animal studies: Two studies had a low risk of bias in all domains [54,55]. Four studies had a high risk of bias in the selection of participants’ domain [56-59]. Three studies had performance bias [56,58,60], four studies had detection bias [56-58,61], two studies had attrition bias [62,63], and two studies had reporting bias [64,65], as summarized in Table 3. Some examples of the bias in these studies would show objectivity. Otherwise, it is just a subjective decision. Everyone should be able to come up with the same result.

**Table 3:** Methodological quality assessment of animal studies using STYCLE assessment framework

Study	Selection bias	Performance bias	Detection bias	Attrition bias	Reporting bias	Other bias	Overall bias
Bourdineaud et al. [54]	Low	Low	Low	Low	Low	Low	Low
Tuhanioğlu et al. [55]	Low	Low	Low	Low	Low	Low	Low
Aliyari et al. [56]	High	High	High	Low	Low	Low	High
Amandokht Saghezchi et al. [57]	High	Low	High	Low	Low	Low	High
El-Maleky and Ebrahim [58]	High	High	High	Low	Low	Low	High
Molina-Montenegro et al. [59]	High	Low	Low	Low	Low	Low	Moderate
Treder et al. [60]	Low	High	Low	Low	Low	Low	Moderate
Doğan et al. [61]	Low	Low	High	Low	Low	Low	Moderate
Bilgici et al. [62]	Low	Low	Low	High	Low	Low	Moderate
Gunes et al. [63]	Low	Low	Low	High	Low	Low	Moderate
Ersoy et al. [64]	Low	Low	Low	Low	High	Low	Moderate
Gupta and Srivastava [65]	Low	Low	Low	Low	High	Low	Moderate

Certainty of Evidence

The GRADE framework emphasizes strengths and limitations in key domains, including methodological limitations, inconsistency, indirectness, imprecision, and publication bias. There was serious concern regarding methodological limitations (RoB), as most of the studies had a high/moderate risk of bias or some concerns. However, the domains of indirectness, imprecision, inconsistency, and publication bias demonstrated no serious concerns, as summarized in Table 4. However, due to serious concerns in the methodology domain, the level of evidence is considered low (Table 4).

**Table 4:** Certainty of evidence assessed according to the GRADE framework Domains include risk of bias, indirectness, imprecision, inconsistency, and publication bias

GRADE domain	Judgment	Concerns	Level of evidence
Limitations in methodology (risk assessment)	Most of the included studies had a high/moderate risk of bias or some concerns according to the methodological risk of bias assessment tool used	Serious	ƟƟ (Low)
Indirectness	Patients and interventions in studies provide direct evidence for the aim of the review	Not serious	
Imprecision	Most of the selected studies performed an appropriate statistical analysis	Not serious	
Inconsistency	Studies did not show any inconsistencies	Not serious	
Publication bias	All of the studies reported negative outcomes; our aim in the study was to report the negative effects of EMFs on living organisms	Not serious	

Results

General Characteristics

As summarized in Table 5, we included a diverse range of studies published from 2017 to 2024, examining the biological impact of EMF exposure across humans, animals, plants, and in vitro models, with varying degrees of relevance to humans. Most of the studies involved animal models [54-65], followed by in vitro studies performed on human and animal cell lines [47-53], and non-RCTs performed on humans [42-46]. For high-frequency studies, reported outcomes were additionally categorized as thermal, non-thermal, or indeterminate, based on the exposure parameters described. Whenever possible, SAR values were extracted and compared with relevant ICNIRP guidelines to contextualize exposure levels. However, SAR was not consistently reported across RF studies, which limits direct cross-study comparisons of exposure intensity.

**Table 5:** Summary of the main characteristics of the 24 studies included in this review ^a^This study [47] investigated biological responses at 3.5 GHz: a frequency relevant to Wi-Fi, 4G, and 5G technologies. Although 5G-specific effects are not the main focus of this review, the study was included because it addresses endpoints within our scope (Wi-Fi, 4G) and fully meets all predefined methodological quality criteria (see Methods/Table 2). The selection of borderline cases was consistently based on these criteria. ^b^Homing rate refers to the percentage of bees that returned to the hive after being displaced (orientation behavior) Wi-Fi: wireless fidelity; EMF: electromagnetic field; SD: standard deviation; ANOVA: analysis of ariance; GSM: global system for mobile communication; PHQ-15: Patient Health Questionaire; ELF: extremely low frequency; RF: radio frequency; HSP-90: heat shock protein-90; VSLM: velocity straight linear motility; CBF: cross-beat frequency; LHD: lateral head displacement; HPMV: homogeneity of progressive motility velocity; N/A: not available (value not reported in the original study)

Study	Classification	Relevance to humans	Methodology (design/model)	Sample size	Frequency (Hz)	Electric field strength	Magnetic flux density	Exposure conditions	Duration	Statistical analysis and study quality	Significance level
Human studies											
Al-Bayyari [42]	Human (males)	Significantly associated with humans using cell phones	Cross-sectional	Experimental group=52, Control group=104	800-2200 MHz	N/A	N/A	Mobile phone and TV (tech. details not specified)	≤1 h/day vs. >1 h/day (not quantified)	Descriptive statistics, Kolmogorov-Smirnov test, Student’s t-test, Pearson’s Chi-square, Fisher’s exact test	<0.05
Boileau et al. [43]	Human (humans, pregnant women)	The study population and exposure reflect everyday mobile phone use, particularly relevant to daily life in pregnant women	Prospective cohort study	The study started with 1,378 records, of which 1,353 cases were included in the final analysis after removing incomplete or unusable data	900–2600 MHz (typical mobile phone frequency ranges; Boileau et al. examined RF exposure from mobile phones, including GSM, 3G, and Wi-Fi)	N/A	N/A	Real-life mobile phone usage during pregnancy	~0.5 h/day (mean, during pregnancy)	Logistic regression	P=0.0374 for >30 min/day usage in relation to growth restriction. P=0.0508 for 15-30 min/day (marginally significant)
Kösek et al. [44]	Human (secretaries in a hospital)	Directly relevant for workplaces with high LF-EMF exposure	Cross-sectional study of hospital secretaries	143 participants	50 Hz (LF-EMF)	N/A	1545.41 ± 224.91 µT	LF-EMF measurements in hospital workplaces with CVS prevalence	~8 h/day (work-related exposure)	Linear mixed-effects models (LMM), generalized additive mixed models (GAMM)	P<0.05 for LF-EMF exposure. P<0.001 for Schirmer test (both eyes). P<0.05 for logistic regression (CVS risk at >1.725 µT)
Szemerszky et al. [45]	Human (cross-sectional study of electromagnetic hypersensitivity)​	Relevant to perceptions of EMF hypersensitivity	Cross-sectional questionnaire study	473 (76.3% women)	Psychological study focused on the perception and self-assessment of electromagnetic hypersensitivity	N/A	N/A	Self-reported EMF exposure from various devices	Not applicable (survey-based study)	Logistic regression and correlation analysis	P=0.001 for PHQ-15. P<0.001 for impact on daily life. P=0.001 for symptom frequency
Yahya et al. [46]	Human (effect of mobile phone radiation on heart rate variability)	The study investigated the effects of mobile phone radiation on heart rate variability in humans	Experimental study on heart rate variability	5	900–2000 MHz (mobile phones)	N/A	N/A	Mobile phone use in normal and vibration mode	Short-term; exact duration not specified	Mann-Whitney U-test, Chi-square tests	P<0.05 for heart rate changes comparing the normal mode and the vibration mode
Chu et al. [47]^a^	Human - in-vitro (human sperm)	High, especially in the context of prolonged mobile phone and Wi-Fi exposure	In vitro study on human sperm samples	9 (4G/5G), 18 (Wi-Fi)	700 MHz-5 GHz. The study mentions 4G, 5G, and Wi-Fi without specifying exact frequencies. These are typical frequency ranges	N/A	N/A	Exposure via iPhone during WhatsApp call (Wi-Fi) and mobile use (4G/5G)	6 h (single continuous exposure)	Mann-Whitney U-test	P=0.030 for total motility. P=0.024 for progressive motility. P=0.003 for viability (WiFi vs. control)
Górski et al. [48]	Human - in vitro (human sperm motility analysis)	Relevant for occupational ELF-EMF exposure	In vitro study on human sperm samples	20 men	50 Hz (ELF-EMF)	1.887 kV/m for the electric component. 1.640 kV/m for the combined electromagnetic component	7.2 µT for the magnetic component. 7.17 µT for the combined electromagnetic component	ELF-EMF was generated in a test chamber with 4 modes: E, M, EM, EM+DS	0.5 h per sample	ANOVA, post-hoc analysis	P=0.02 to p=0.03 for VSLM. P<0.001 for CBF; no significant changes for LHD and HPMV
Animal studies											
López-Martín et al. [51]	Animal (Sprague-Dawley rats)	Relevant for Wi-Fi exposure	In vivo experiment with female rats	42 rats (21 per group)	2.45 GHz	0,040.28 kv/m - 0,080.56 kv/m	N/A	RF exposure in the GTEM chamber with a uniform field setup	0.5 h, single exposure	ANOVA, multiple comparison tests	P<0.05 for calcitonin-positive cells at 3 and 12 weeks. P<0.001 for co-localization of HSP-90 and calcitonin
Bourdineaud et al. [54]	Animal (Eisenia fetida)	The study examined the link between prenatal mobile phone use and fetal growth in humans	Animal study	8/treatment group	900 MHz	10, 23, 41, 120 V/m	0.3, 1.4, 4.2, 38.2 W/m	Mobile phone	2 h	Mann-Whitney U-test, t-test, qRAPD	P<0.05
Tuhanioğlu et al. [55]	Animal (Wistar rats)	Indirect relevance for medical PMF applications	In vivo study on Wistar-Albino rats	12 (6 per group)	40 Hz (pulsed magnetic fields)	0.0006 kV/m (electric)	1500 µT	Helmholtz coil system with Faraday cage	30 h (1 h/day for 30 days)	ANOVA, Tukey’s post-hoc test	P<0.05 for hearing thresholds at 5714 Hz and 8000 Hz, P<0.001 for apoptosis at Caspase-3, Caspase-9, and TUNEL
Aliyari et al. [56]	Animal (Rhesus macaques)	Potentially significant, particularly for individuals living near high-voltage towers	Experiment with Rhesus macaque: one control and one exposed animal	2	50 Hz. The study mentions high-frequency EMFs. This is a typical frequency	3kv/m (electric)	N/A	Simulated high-voltage electric towers in a controlled environment	120 h total (4 h/day × 30 days)	Descriptive and comparative analysis	Increased adrenaline and blood sugar levels: elevated levels were observed following EMF exposure, but no specific p-values were provided
Amandokht Saghezchi et al. [57]	Animal (NMRI mice)	Potentially relevant for pregnant women exposed to Wi-Fi	Experimental study on NMRI mice	21 (3 groups with 7 animals each)	2.4 GHz	N/A	N/A	Wireless Router (CISCO, EA6300V1, China), 20–30 cm	84 h (4 h/day for 21 days)	One-way ANOVA, LSD test (precise for small groups)	P-values for significant effects: p< 0.001 for bone volume. P<0.01 for cartilage volume and gene expression of osteocalcin and RUNX2
El-Maleky and Ebrahim [58]	Animal (albino rats)	High relevance for long-term phone users	Animal study on male albino rats	24 rats were used, divided into 3 groups with 8 animals each	890–915 MHz (GSM)	Not directly stated (SAR=0.96 W/kg)	N/A	GSM phone in "on-call" mode placed 0–1 cm from rat cages	15–180 h (0.5–1 h/day for 1–6 months)	Descriptive and regression analysis	P<0.01 for serum hepcidin. P<0.001 for TLC. P<0.01 for serum ferritin
Molina-Montenegro et al. [59]	Animal - insects (honeybees)	Indirect relevance via agriculture and ecosystem dependence	Combined field and lab study on bees and pollination	72 cages (36 close active, high voltage lines, 36 controls)	EMFs generated by high-voltage lines at 50 Hz	N/A	1.5 µT in inactive high-voltage power lines (EMF-off). 9.47 µT (± 0.21 SD) in active high-voltage power lines (EMF-on)	Bees exposed near high-voltage towers (5-100m distance) and in solenoid-based lab setups	~2 h total (5 min/day for 25 days)	Generalized linear models (GLMs)	P<0.0001 for Hsp70 expression. P<0.05 for bee visits. P<0.05 for seed production (natural pollination at active hives)
Treder et al. [60]	Animal - insects (honeybee colonies)	Indirect relevance through pollination and agriculture	Long- and short-term studies on bee behavior	8 colonies (long-term), 9 (short-term)	2.45 GHz, 5.8 GHz	N/A	N/A	Controlled lab and field trials with bee colonies	120 h total (2 h/day, 5 d/week for 12 weeks)	Mixed models and survival analysis	P=0.0064^b^ for homing rate (long-term). P=0.102 for longevity. P=0.862 for brood development
Doğan et al. [61]	Animal (Wistar rats)	The authors examined the physiological responses of rats to high-voltage lines	Experimental study on Wistar rats	A total of 64 Wistar albino rats were used, divided into 8 groups	50 Hz	0,0803 kV/m	2480 µT	High voltage (10 kV) generated by transformers, continuous exposure	208–416 h (8 h/day for 26–52 days)	Mann-Whitney U-test, t-test (basic but suitable for small samples)	P<0.05 for odontoblast degeneration, inflammatory cell infiltration, and vasodilation/hemorrhage (ELF-EMF exposure)
Bilgici et al. [62]	Animal (Wistar rats)	Relevant for long-term Wi-Fi exposures. The authors mention potential long-term effects that could be relevant for humans	Experimental study in Wistar rats	22	2.45 GHz	0.00368 ± 0.00036 kV/m	0.1-1 µT (based on typical values at this frequency)	Controlled Wi-Fi exposure in plexiglass cages	30 h (1 h/day for 30 days)	Mann-Whitney U-test, t-test	P<0.05 for IL-6, CRP, and spermatogenesis/histopathology
Gunes et al. [63]	Animal - insects (Drosophila melanogaster)	Relevant for mobile phone signals typically used in 2G–4G networks; thus, of potential relevance to human exposure	Experimental study with Drosophila larvae under SMART assay	11 groups (including control)	900 MHz, 1800 MHz, 2100 MHz	0.0352 kV/m - 0.041 kV/m	N/A	RF-EMF in an anechoic chamber with a monopole antenna setup	2–6 h/day for 2 days	Parametric and non-parametric tests	P<0.05 for 900 MHz at 2, 4, and 6 hours. P<0.05 for 2100 MHz at 4 and 6 hours, no significant change at 1800 MHz
Ersoy et al. [64]	Animal (Sprague-Dawley rats)	Relevant for long-term ELF-EMF exposure	Animal study on Sprague-Dawley rats	The total number of animals is 35, but the group breakdown is: sham (n=15), EMF-28 (n=10), EMF-42 (n=10)	50 Hz	N/A	3000 µT	Helmholtz coil at 3 mT (50 Hz)	140–180 h total (4 h/day × 5 d/wk × 7–9 wk)	Logistic regression and survival analysis	P<0.05 for FSH, LH. P<0.001 for testis weight and GSH (glutathione)
Gupta and Srivastava [65]	Animal (chickens)	Relevant for oxidative stress and reproductive health in humans	Animal study with immature male Gallus gallus domesticus	14 animals (7 control, 7 exposed)	2.45 GHz (microwave)	N/A	N/A	Ruckus R310 Wi-Fi router in continuous mode within an octagonal chamber	60 h total (2 h/day × 30 days	One-way ANOVA, Tukey’s post-hoc test	P<0.01 for body weight. P<0.001 for testis weight and volume. P<0.05 for MDA, H2O2, and histopathological changes
Plant and in vitro studies											
Górski et al. [49]	Human - in vitro (fibroblast and prostate cancer cell cultures)	Relevant for cancer research	In vitro study with fibroblasts and prostate cancer cells	5000 Zellen pro Well, in 96-Well-Platten	2.4 GHz (Wi-Fi/Bluetooth)	0.263 kV/m	N/A	RF-EMF with Bluetooth antenna in a controlled chamber	24-72 h	Kruskal-Wallis test, post-hoc Dunn test	P<0.05 for fibroblasts after 24-48 hours. P<0.01 for PC-3 cells after 48-72 hours
Lefebvre et al. [50]	In vitro (PC-12 neuronal cell cultures)	Potential for EMF in neuro-regeneration	In vitro study with PC-12 cell lines	24 plates (6 per group) or: In fact, 18 out of 24 samples were fully analyzed due to some technical limitations	7.8 Hz (Schumann-frequency). 29.3 Hz, 30.3 Hz, 71 Hz, 79.1 Hz	N/A	1 µT	Physiological EMFs in Helmholtz coils with a sine-wave generator	0.67 h (single exposure)	Regression analysis, analysis of variance	P<0.05 for LF-EMF exposure. P<0.001 for Schirmer test (both eyes). P<0.05 for logistic regression (CVS risk at >1.725 µT)
Sukhov et al. [52]	Plant (wheat and pea seedlings)	Indirect relevance for agriculture and crop productivity	In vitro with wheat and pea plants	Wheat: 30, pea: 9 (short); 6 (long)	7.8 Hz, 14.3 Hz, 20.8 Hz	N/A	18 µT	Schumann resonances with sinusoidal current modulation	0.5 h (short-term); 9 days (long-term)	Chi-square, t-tests	P<0.05 for NPQ, t1/2(ΦPSII), and NPQS in wheat under 14.3 Hz treatment
Echchgadda et al. [53]	In vitro (primary hippocampal neurons)	Relevant for environments with prolonged RF-EMF exposure	In vitro neuronal study	12 plates	3.0 GHz	0.137 kV/m	N/A	In vitro exposure of primary hippocampal neurons in a closed chamber	1 h (single continuous exposure)	Kruskal-Wallis, Mann-Whitney U-test	P=0.03 for action potential amplitude and resting potential. P<0.001 for intracellular Ca²⁺

A wide range of frequencies was used to assess their negative impact, ranging from extremely low frequencies (40 and 50 Hz) [55,61] to high frequencies (2.45 GHz) [51,65]. Likewise, the minimum field strength was 0.0006 kV/m [55], and the maximum was 3 kV/m [56]. When reported, specific absorption rate (SAR) values were included (e.g., 0.14 W/kg [62], 0.96 W/kg [58]), and studies were categorized according to their primary exposure mechanism as thermal or non-thermal, with the majority investigating non-thermal effects.

Magnetic flux density also varied from ≤1 µT [50,59,62] to >1000 µT [44,55,64]. The most targeted endpoints identified were heart rate variability, sperm mortality, behavioral changes, and oxidative stress, with a significance level of 0.05 for most of the studies (Table 5). Methodological heterogeneity was also evident in statistical analyses, which ranged from simple comparisons to complex regression models, complicating data synthesis. Moreover, study quality varied, with limitations often including small sample sizes, short exposure durations, lack of long-term follow-up, and limited external validity.

Outcomes

Table 6 summarizes the selected studies investigating the physiological and biological impact of EMFs from various sources, such as mobile phones, Wi-Fi, and ELF/RF radiations. The impact of these EMFs was evaluated on a multitude of biological phenomena, including sperm quality [42,61,62], hormonal levels [61,62], behavioral changes [56,59-61], oxidative stress [56,58,61,62,65], DNA modification [49,54,56,61,63], cardiac and neurological functions [46,56,61], hearing [55,58], reproductive development [57,61,62], and behavioral/physiological changes in plants and insects [46,52,59,60,63].

**Table 6:** Summary of outcomes The following grades indicate the strength and reliability of the evidence: Grade A: strong evidence, high-quality study design (e.g., low risk of bias, adequate sample size, replicable findings); Grade B: moderate evidence, acceptable methodological rigor, but with some limitations (e.g., small sample size, minor bias); Grade C: weak evidence, significant methodological concerns (e.g., high risk of bias, inconsistent findings); Grade D: very weak or inconclusive evidence, serious methodological flaws or insufficient data ECG: electrocardiogram; Wi-Fi: wireless fidelity; EMF: electromagnetic field; PHQ-15: Patient Health Questionaire; ELF: extremely low frequency; RF: radio frequency; HSP-90: heat shock protein-90; VSLM: velocity straight linear motility; CBF: cross-beat frequency; LHD: lateral head displacement; HPMV: homogeneity of progressive motility velocity; N/A: not available; MDA: malondialdehyde; DNA: deoxyribonucleic acid; VEGF: vascular endothelial growth factor; IL: interleukin; TV: television; CRP: C-reactive protein; RT-PCR: reverse transcription ploymerase chain reaction; Hb: hemoglobin; MCV: mean corpuscular volume; MCH: mean corpuscular hemoglobin; SOD: superoxide dismutases; IGF1: insulin-like growth factor 1; FSH: follicle-stimulating hormone; LH: luteinizing hormone

Study	Outcome measures	Effect size	Key findings	Strengths and limitations	Conclusions	Funding source	Grade
Al-Bayyari [42]	Semen quality	Decreased	Sperm quality reduced with TV/mobile use	Strength: clinic sample, clear measures. Limitation: self-report use, motives unassessed	Negative association; recall bias	N/A	A
Bourdineaud et al. [54]	DNA stress genes	Increased	DNA modification observed	Strength: mechanistic biomarker focus. Limitation: no other outcomes; no dose-effect	EMF affects stress-response genes	N/A	A
Doğan et al. [61]	Tooth histopathology	Increased	Pulp damage; melatonin protective	Strength: controlled, detailed histology. Limitation: o long-term data	ELF-EMF causes dental changes	N/A	A
Bilgici et al. [62]	IL-6, CRP, histology	Increased	Inflammation and testicular damage	Strength: precise analysis. Limitation: no systemic markers; small N	Wi-Fi damages testicular tissue	N/A	B
Yahya et al. [46]	HRV (ECG)	Increased short-term HRV	Short-term heart rate variability changes	Strength: realistic exposure. Limitation: small N; acute only	Short-term HRV effect	N/A	C
Tuhanioğlu et al. [55]	Hearing, apoptosis	Increased	Cochlear damage and apoptosis	Strength: standardized DPOAE. Limitation: limited to the cochlea; small N	Hearing and cell changes after EMF	N/A	B+
Amandokht et al. [57]	Bone, genes (RT-PCR)	Decreased	Bone volume and gene expression reduced	Strength: stereology, molecular detail. Limitation: short-term, localized	Prenatal RF impairs bone and genes	Shahid Beheshti University of Medical Sciences	B
El-Maleky et al. [58]	Iron metabolism, hepcidin	Decreased	Iron reduced; ferritin and Hb affected	Strength: controlled, long exposure. Limitation: no field strength; no follow-up	EMF alters iron metabolism	N/A	A
Szemerszky et al. [45]	IEI-EMF surveys	Increased	Reported symptoms and diagnosis complexity	Strength: large sample, refined criteria. Limitation: self-report, no objective exposure	Diagnosis needs multiple criteria	Supported by the Hungarian National Scientific Research Fund, the János Bolyai Research Scholarship, and the New National Excellence Program of the Ministry of Human Capacities​	B
Boileau et al. [43]	Fetal growth, birth outcomes	Increased	FGR risk with >30 min/day phone use	Strength: covariate adjustment. Limitation: self-report, no exposure data	Phone use linked to FGR risk	N/A	A
Górski et al. [48]	Sperm motility	Decreased	Reduced motility under magnetic/EM exposure	Strength: calibrated, comparative. Limitation: in vitro only; no long-term data	ELF-EMF reduces sperm motility	N/A	A
Gunes et al. [63]	Genotoxicity	Increased	Mutant clones increased at 900/2100 MHz	Strength: controlled assay. Limitation: insect model; limited relevance	RF-EMF may induce genotoxicity	N/A	A
López-Martín et al. [51]	Thyroid, cell markers	Increased	Calcitonin hyperplasia and HSP changes	Strength: molecular, controlled. Limitation: female rats only; short-term	RF triggers thyroid stress	Supported by Spanish and EU research projects	B+
Górski et al. [49]	Cell viability	Decreased fibroblast viability; increased cancer activity	Fibroblast activity↓; Ca cells↑	Strength: detailed exposure, multimodal. Limitation: in vitro, no SAR, few lines	RF-EMF shifts cell activity	Supported by the Polish Ministry of Science and Higher Education	B
Sukhov et al. [52]	Photosynthesis, NPQ	Reduced NPQ; altered electron transport	NPQ reduction, E transport altered	Strength: short/long-term, detailed. Limitation: lab only, limited species	Low-frequency EMFs affect photosynthesis	Supported by the Russian Federation government funding	A-
Gupta et al. [65]	Repro. markers, oxidative stress	Decreased/increased	Testis damage, MDA↑, GSH↓	Strength: detailed markers, histology. Limitation: animal model; 30-day max	MW/EMF disrupts the testis and redox	Supported by the Indian University Grants Commission and Dr. Harisingh Gour Central University	A
Aliyari et al. [56]	Behavior, blood cells, MRI	Increased/Decreased	WBC↑, RBC↓, stress markers↑	Strength: MRI, multiple biomarkers. Limitation: small N, short-term	HV-EF elevates stress; alters blood	Supported by Baqiyatallah Neuroscience Research Center and Amirkabir University	B
Echchgadda et al. [53]	Neuron excitability, Ca2+	Increased	Synaptic activity and Ca2+ increased	Strength: electrophysiology. Limitation: in vitro only, acute	RF-EMF increases neuron activity	Supported by the United States Air Force Research Laboratory and General Dynamics Information Technology	A
Chu et al. [47]	Sperm motility/viability	Decreased	Wi-Fi impairs motility; 4G/5G not	Strength: calibrated, multi-frequency. Limitation: small N; no in vivo	Wi-Fi impairs sperm; 4G/5G not	N/A	B
Ersoy et al. [64]	Testis, hormones, oxidative	Decreased	FSH, LH↓; testis damage	Strength: detailed, long-term. Limitation: rats only; puberty not assessed	ELF-EMF harms the testis and hormones	Supported by Dokuz Eylul University Scientific Research Foundation	A
Molina-Montenegro et al. [59]	Bee visits, HSP70, seed prod.	Decreased/increased	Bee stress↑; plant fitness↓	Strength: field + lab. Limitation: locale only; 6 weeks	EMF disrupts bees and pollination	Universidad de Talca, Chile; Chilean institutions; American Association for the Advancement of Science	A
Treder et al. [60]	Bee homing, brood	Decreased	Homing↓; brood unaffected	Strength: controlled, innovative. Limitation: no overwintering data	RF as bee sublethal stressor	Funded by Baden-Württemberg Ministry for Agriculture and Rural Areas, Germany	A-
Kösek et al. [44]	CVS, LF-EMF	Increased	CVS and dry eye syndrome increased	Strength: workplace, validated scales. Limitation: cross-sectional, single-site	LF-EMF increases CVS/dry eyes	Supported by Çukurova University	B
Lefebvre et al. [50]	Neurite growth, electrophysiology	Neurite outgrowth ± (frequency-dependent)	Outgrowth modulated; Akt↑	Strength: patch clamp, detailed patterns. Limitation: in vitro, few cell lines	Patterned EMF modulates neurites	N/A	A

Inconsistencies in the outcomes also persist, particularly concerning long-term exposure effects. While earlier studies reported minimal changes, more recent research suggests the potential for cumulative impacts. These discrepancies are likely attributable to methodological differences, such as variations in exposure duration, frequency, and study designs. For instance, López-Martín et al. [51] identified thyroid cell homeostasis disruptions following a single 30-minute exposure to 2.45 GHz at a power density of 0.18 mW/cm² (see Table 1 for units). Similarly, Bilgici et al. [62] observed oxidative stress, a 35% reduction in sperm motility, and necrotic testicular tissue in rats exposed to 2.45 GHz for 60 minutes per day over 30 consecutive days (SAR: 0.14 W/kg). In humans, Górski et al. [48] reported reduced motility after EMF exposure. In zebrafish embryos, behavioral patterns were altered after a 24-hour exposure to 900 MHz. These additional observations, such as altered neuronal excitability [53] and pollination behavior in honeybees [59], further emphasize the need to investigate frequency-specific impacts on biological systems. For a complete overview of all exposure parameters and biological effects (Table 5).

The consistent observation of biologically relevant EMF effects across diverse study designs supports the hypothesis that EMF exposure may disrupt physiological processes across species. This becomes evident when comparing ecological disruptions in honeybees [59] with cellular-level findings [50] or oxidative stress effects in mammals [58], which are difficult to reconcile with one another. A major limitation across studies was their methodological diversity, the absence of standardized exposure metrics, such as field strength normalized to body mass (kV/m per kg), and inconsistencies in exposure frequency and duration. This lack of standardization impedes cross-species comparisons and dose-response assessments. Furthermore, differences in exposure protocols and biological models add to this variability.

The wide variation in exposure durations-from single exposures [51] to prolonged exposures over 30 days [61], along with the absence of standardized metrics, complicates data synthesis. These factors hinder comparability and limit the reliability of meta-analyses. This variability also limits extrapolation to chronic real-life exposures that span months or years, particularly in humans. Without long-term studies, it remains uncertain whether short-term effects accumulate, diminish, or trigger delayed physiological responses, highlighting the need for long-term research. This not only complicates comparability and hampers reliable meta-analysis, but also raises broader epistemological concerns. As Feest [66] emphasized within the discussion on solutions for the replication crisis, the aim is not simply to accumulate identical results but to explore diverse experimental designs that converge on shared biological hypotheses.

Some studies nonetheless provided mechanistic [R 21] [SD2] insights, such as indications of oxidative stress, mitochondrial dysfunction, or altered gene expression. Here, “mechanistic insights” refer to experimental indications of specific biological pathways affected by EMF exposure-such as increased markers of oxidative stress (e.g., elevated reactive oxygen species), signs of mitochondrial dysfunction (e.g., reduced ATP production, disrupted membrane potential), or altered gene expression (e.g., changes in levels of stress response genes). For instance, Górski et al. [48] observed reduced sperm motility in humans, potentially associated with oxidative stress and membrane damage, although no direct causality was established. Lefebvre et al. [50] reported mitochondrial dysfunction, ROS formation, and altered gene expression, pointing to intracellular stress responses. López-Martín et al. [51] demonstrated altered calcitonin-dependent activity and HSP-90 modulation in parafollicular thyroid cells, suggesting EMF-induced protein expression changes and endocrine disruption. These findings indicate that EMFs might affect distinct biological targets such as proteins (e.g., heat shock proteins), organelles (e.g., mitochondria), and hormonal axes (e.g., thyroid function).

In contrast, Boileau et al. [43] and Chu et al. [47] described effects on behavior and neurological outcomes without exploring underlying cellular or molecular pathways. This inconsistency in mechanistic detail-ranging from studies identifying intracellular pathways to those only reporting behavioral effects-emphasizes the need to systematically investigate the biological processes underlying EMF-related changes.

Table 6 summarizes key characteristics, principal results, and methodological considerations for all studies included in this review.

Discussion

This discussion is structured into three sections: First, we summarize the key biological effects associated with EMF exposure reported across the reviewed studies. Second, we evaluate methodological limitations and potential sources of bias affecting the reliability and comparability of results. Finally, we identify current research gaps and outline directions for future investigations. The categorization of EMF effects as “negative” or “positive” should not be viewed as absolute. As Paracelsus noted, “the dose makes the poison”-this principle is quantified in radiation biology by considering not only the presence of a field, but also its intensity, exposure duration, and the specific biological context. Similarly, the effects of EMFs depend on these parameters, emphasizing that their impact is not inherently harmful or benign.

This review adopts a cautious perspective, focusing on reported detrimental outcomes while acknowledging the complexity of such classifications. This systematic review summarizes studies on the mainly detrimental impacts of EMFs across a wide range of biological systems and living organisms. Interpretation of the results requires critical consideration of study quality and heterogeneity. Most experiments were conducted in vitro or on animal models, often with short exposure durations and varied parameters (e.g., frequency, intensity, wave type). Many studies exhibited moderate to high risk of bias, and standardized exposure metrics were frequently missing, limiting comparability and generalizability. The following section summarizes the most frequently reported biological effects of EMF exposure. Despite methodological limitations, several patterns emerged: EMFs primarily affected oxidative stress mechanisms, inflammatory responses, and disrupted cellular, physiological, and ecological processes [56,58-63]. These findings underscore the systemic complexity of EMF interactions.

Similar to effects observed in radiation biophysics, oxidative stress appears to be a central mechanism. While ionizing radiation causes oxidative stress via direct ionization and free radical formation, non-ionizing EMFs likely induce it indirectly, through mitochondrial dysfunction, calcium influx, or altered gene expression [67]. Over time, such mechanisms may lead to oxidative damage to DNA, proteins, and lipids. Consistent with this, Amiri et al. [68] observed correlations between mobile phone use and blood pressure fluctuations in a large cohort. However, the reliance on self-reported data and inconsistent study designs highlights the need for more rigorous research. In contrast, Elmas [69] found no conclusive effects of mobile phone exposure on cardiovascular or general health outcomes. Such discrepancies may reflect variations in EMF intensity, exposure duration, or individual susceptibility. Importantly, EMFs are also used therapeutically, for instance, in treating myocardial ischemia. While several in vitro studies reported oxidative damage and impaired cell viability, variability in endpoints and models limits definitive conclusions about cellular mechanisms.

Given the theoretical sensitivity of developing biological systems-particularly in children-even low-level, long-term EMF exposure may warrant closer investigation. Some researchers also speculate that EMF-induced stress could trigger adaptive or immune-related responses, though current evidence is inconclusive. These considerations emphasize the need for well-designed long-term studies to explore cumulative and age-dependent effects. Most studies examined short-term exposures (30 minutes to 30 days; e.g., López-Martín et al. [51]; Doğan et al. [61]), leaving long-term impacts largely unexplored. None of the included studies attempted to extrapolate short-term results to longer periods by means of linear regression models or dose-time modeling (e.g., dose per kg per time). All reported outcomes are restricted to the specific exposure durations actually tested. Consequently, inferring chronic or cumulative effects from these short-term studies remains problematic and highlights a major research gap. For instance, observed disruptions in honeybee behavior and plant photosynthesis raise concerns about ecological consequences, including pollination, crop yields, and ecosystem stability.

Long-term ecological studies are necessary to assess delayed or cumulative biological responses, especially in critical species such as pollinators, crops, and apex predators. The existing literature reveals several methodological limitations. A key challenge is the variability in exposure protocols-frequency, intensity, and duration-which impedes comparability and limits generalizability. Outcome reporting also varies widely. Although this review applied a comprehensive search strategy, inconsistencies may reflect limitations in keyword-based selection and indexing. The lack of standardized exposure metrics further restricts comparability. Additionally, the possibility of missed studies due to publication lag, database limitations, or restricted indexing cannot be fully excluded, which may have led to the omission of relevant research despite comprehensive search efforts. Some meta-analyses suggest that industry-funded studies are statistically less likely to report adverse outcomes, indicating potential bias [30]. However, as Feest [66] emphasizes, such patterns require careful contextualization and should not be interpreted as universal.

Diverse methodologies and funding sources may shape results in complex ways, warranting critical examination rather than simplistic categorization. Because of the fragmented nature of existing findings, no definitive conclusions can be drawn regarding critical exposure parameters such as field strength or magnetic flux density. In particular, for low-frequency EMFs, controversies persist regarding both the mechanisms of action and the reproducibility of reported biological effects. While some studies suggest potential impacts on cellular processes, the clinical significance of these findings remains uncertain and warrants cautious interpretation. The lack of standardized reporting hinders dose-response analyses and cross-study comparisons. Differences in experimental design-e.g., acute versus chronic exposure-further complicate interpretation. Additionally, most animal studies included in this review were limited to 30 days of exposure, offering little insight into chronic effects. Methodological bias remains a significant challenge.

To ensure this review’s validity, we critically assessed biases within and across studies, with attention to data gaps and inconsistencies. The heterogeneity in study design, exposure conditions, and measured outcomes complicates synthesis. Finally, we identify key research gaps. Long-term studies are urgently needed to evaluate cumulative and long-term health risks, as short-term studies may overlook delayed or chronic responses. Furthermore, the present review excluded 5G-related studies due to the limited availability of high-quality experimental research within the 2017-2024 time frame. While this approach improved methodological consistency, it also restricts the applicability of our conclusions to emerging high-frequency millimeter-wave technologies, warranting future investigation as robust datasets become available. Future research should also account for baseline health factors such as age, health status, and pre-existing conditions, which may significantly influence biological responses. 

These findings should be interpreted in the context of current consensus statements from major health organizations, such as the World Health Organization and the International Commission on Non-Ionizing Radiation Protection, which generally conclude that, within established exposure limits, non-ionizing EMFs are unlikely to cause adverse health effects. Nonetheless, the present review identifies specific domains-particularly regarding long-term and cumulative exposures-where evidence remains limited and further high-quality research is warranted. Incorporating these variables could support the development of more accurate and population-specific exposure guidelines.

## Conclusions

The rapid evolution of wireless communication technologies continues to introduce new applications and expand into higher frequency ranges. This review identifies many experimental studies reporting the biological effects of EMFs on humans, animals, and plants. These include changes in fertility parameters, cellular responses associated with oxidative stress, developmental outcomes, behavioral alterations, and cognitive effects. Our findings highlight why it is important to take a closer, structured look at how EMF exposure might affect both human health and the environment. This review focused on reported biological effects, without implying universality or disregarding studies that observed neutral or application-related outcomes. The diversity in reported results highlights the need for cautious interpretation and context-specific analysis.

Significant research gaps remain. Short-term effects have drawn much attention in past studies, but the more pressing question (what happens over time?) remains largely unanswered, especially concerning people and species highly sensitive to their environment. Additionally, a lack of standardized exposure protocols and inconsistent reporting of critical parameters such as field strength and frequency limit reproducibility and hampers the formulation of robust exposure guidelines. Addressing these challenges means committing to research that is not rushed, not vague, and not influenced by unclear funding. Long-term, transparent, and solid work is the only way forward. These should include clearly defined exposure parameters, attention to population-specific vulnerability factors (e.g., age, baseline health), and ecological relevance. As seen historically, public concern and scientific controversy can foster critical inquiry and the development of evidence-based safety standards. A coordinated effort is needed to enhance our understanding of EMF interactions with biological systems and to support informed decision-making in public health initiatives and policy.

## Appendices

**Table 7:** Literature search conducted using various databases

Databases	Search terms
MEDLINE	(("electromagnetic waves"[All Fields] OR "electric magnetic field"[All Fields] OR "electromagnetic radiations"[All Fields] OR (("schumann"[All Fields] OR "schumann s"[All Fields]) AND ("epidemiology"[MeSH Subheading] OR "epidemiology"[All Fields] OR "frequency"[All Fields] OR "epidemiology"[MeSH Terms] OR "frequence"[All Fields] OR "frequences"[All Fields] OR "frequencies"[All Fields])) OR "schumann resonance"[All Fields] OR "EMF"[All Fields]) AND ("living beings"[All Fields] OR "living organisms"[All Fields] OR "humans"[All Fields] OR "animals"[All Fields] OR "cells"[All Fields] OR "biological systems"[All Fields]) AND ("negative effects"[All Fields] OR "negative outcome"[All Fields] OR "detrimental impact"[All Fields] OR "adverse effects"[All Fields]))
The Cochrane Library	((“electromagnetic waves” OR “electric magnetic field” OR “electromagnetic radiations” OR “schumann frequency” OR “schumann resonance” OR “EMF”)):ti,ab,kw AND ((“living beings” OR “living organisms” OR “humans” OR “animals” OR “cells” OR “biological systems”)):ti,ab,kw AND ((“negative effects” OR “negative outcome” OR “detrimental impact” OR “adverse effects”)):ti,ab,kw
Scopus	(“electromagnetic waves” OR “electric magnetic field” OR “electromagnetic radiations” OR “schumann frequency” OR “schumann resonance” OR “EMF”) AND (“living beings” OR “living organisms” OR “humans” OR “animals” OR “cells” OR “biological systems”) AND (“negative effects” OR “negative outcome” OR “detrimental impact” OR “adverse effects”)
